# The loss of profilin1 is catastrophic to podocytes

**DOI:** 10.1172/JCI175594

**Published:** 2023-12-15

**Authors:** Sandeep K. Mallipattu

**Affiliations:** Division of Nephrology, Department of Medicine, Stony Brook University, Stony Brook, New York, USA. Renal Section, Northport VA Medical Center, Northport, New York, USA.

## Abstract

Profilin1 belongs to a family of small monomeric actin-binding proteins with diverse roles in fundamental actin-dependent cellular processes required for cell survival. Podocytes are postmitotic visceral epithelial cells critical for the structure and function of the kidney filtration barrier. There is emerging evidence that the actin-related mode of cell death known as mitotic catastrophe is an important pathway involved in podocyte loss. In this issue of the *JCI*, Tian, Pedigo, and colleagues demonstrate that profilin1 deficiency in podocytes triggered cell cycle reentry, resulting in abortive cytokinesis with a loss in ribosomal RNA processing that leads to podocyte loss and glomerulosclerosis. This study demonstrates the essential role of actin dynamics in mediating this fundamental mode of podocyte cell death.

## Podocyte detachment and cell death

Podocytes are postmitotic visceral epithelial cells that cover the glomerular basement membrane. Collectively with the glomerular endothelial cells and basement membrane, podocytes constitute the kidney filtration barrier. Podocyte injury with ensuing loss is the major determinant of progressive glomerular disease. A large body of evidence demonstrates that a multitude of mechanisms could portend the eventual demise of podocytes, extending from apoptosis, anoikis, entosis, dysregulated autophagy, pyroptosis, senescence, and necroptosis (1–3). Regardless of the etiology contributing to podocyte loss, there is ultimately a collapse in the actin cytoskeleton and loss of terminal differentiation markers leading to eventual podocyte detachment. Because these cells are in G_1_ arrest after development with no capacity for self-renewal (4), their loss is devastating to the structure and function of the glomerular filtration barrier. In glomerular diseases, such as idiopathic collapsing glomerulopathy and HIV-associated nephropathy, there is an increase in the mitotic cell cycle proteins cyclins A, B1, and D1 and a decrease in the cell-dependent kinase inhibitors p21, p27, and p57, resulting in the loss of terminal differentiation markers (5, 6). However, podocytes are unable to successfully complete mitosis as a result of abortive cytokinesis, leading to cell death.

During mitosis, proper actin-microtubule-dependent segregation of duplicated chromosomes is tightly regulated to maintain genomic stability. Originally observed in the late 1930s (7) and later coined in 1986 (8), mitotic catastrophe ensues after a loss of surveillance systems involving chromosomal breaks, karyokinesis, and cytokinesis due to the incomplete formation of mitotic spindles, which presents as micronucleation, multinucleation, or irregularly shaped nuclei (2, 9, 10). Previous studies report mechanisms involving MDM2 inactivation of p53-mediated cell cycle arrest, activation of Notch signaling, loss of Krüppel-like factor 4 triggering dysregulated STAT3 activation, and reduction in myeloid-derived growth factor signaling, all of which contribute to mitotic catastrophe of podocytes (11–13). Regardless of the inciting event, altered actin dynamics ultimately contribute to abortive cytokinesis in mitotic catastrophe. Interestingly, a recent study demonstrated that a substantial proportion of podocytes lost in the urine in patients with diabetes mellitus exhibit features of mitotic catastrophe, broadening its potential impact in glomerular diseases (14).

## Essential role of profilin1 in actin dynamics

**Figure 1:**
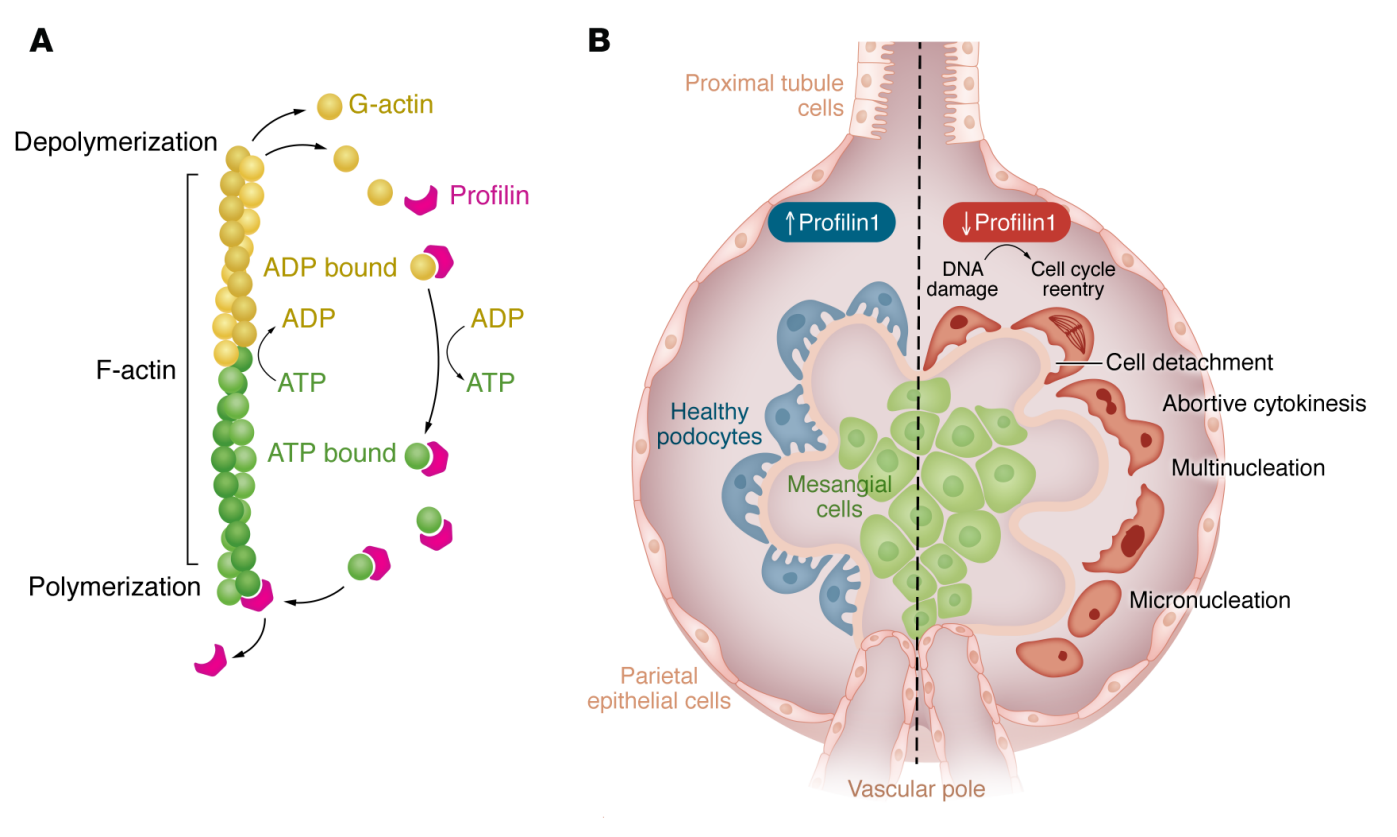
Profilins have a critical role in actin dynamics and preventing mitotic catastrophe. (**A**) Profilins have a role in sequestering globular actin (G-actin) monomers and replenishing the ATP-actin monomer pool (dark green), so G-actin monomers can be assembled into F-actin in a concentration-dependent manner. During actin polymerization, ATP-actin is assembled into F-actin, and it is subsequently hydrolyzed by ATPase, leading to the conversion of ADP-actin. Actin depolymerization occurs at the end of the filament with the release of ADP-actin. (**B**) Profilin loss induces dsDNA damage, triggering chromatin instability and subsequent cell cycle reentry in podocytes. Subsequently, this leads to defective cytokinesis, cell detachment, and eventual mitotic catastrophe.

Profilins are a family of small (approximately 15 kDa), highly conserved, actin monomer-binding proteins critical for actin-dependent cellular processes, such as motility, signal transduction, DNA repair, cell trafficking, cytokinesis, and proliferation in eukaryotic cells (15). Profilins were initially observed for their role in sequestering actin monomers and later reported to have critical functions in the assembly of globular actin monomers into F-actin in a concentration dependent manner (16) (Figure 1). In mammals, four isoforms constitute the profilin family, with profilin2, profilin3, and profilin4 demonstrating tissue specificity compared with the more well-studied and ubiquitously expressed profilin1 (Pfn1) (17). Initially described in 1977, Pfn1 is essential for cell survival owing to its fundamental role in regulation of actin cytoskeleton via interactions with actin binding proteins and proline repeat domains (18–20). In addition to its ubiquitous expression, Pfn1 is expressed in all stages of embryonic development, and loss of Pfn1 is embryonically lethal in mice (21), thereby requiring generation of inducible and conditional knockout models to study its role in specific cell types after development. Furthermore, Pfn1 is critical for the coordination of actin and microtubule network in axon growth and regeneration postnerve injury (22). In addition to its role in multiple cancers and cardiovascular disease, Pfn1 mutations have also been implicated in amyotrophic lateral sclerosis (17).

During mitosis, Pfn1 interacts with the actin binding protein formins to increase actin filament elongation rates during cytokinesis (23). Consequently, the loss of Pfn1 is embryonically lethal due to defective cytokinesis and, in some cell types, also associated with malformed actomyosin contractile rings during late mitosis (21). More recently, Bottcher et al. observed that Pfn1 was necessary to generate sufficient traction forces required for abscission during late cytokinesis (24).

## The role of Pfn1 in podocyte biology

In this issue of the *JCI*, Tian, Pedigo, and colleagues provide multiple lines of evidence for the requisite role of Pfn1 in preserving podocyte health and, specifically, its role in preventing cell cycle reentry and eventual mitotic catastrophe (25) (Figure 1). First, mice with podocyte-specific deletion of Pfn1, while normal at birth, demonstrated an increase in albuminuria and worsening kidney function starting at 6 weeks of age, which led to progressive weight loss and eventual death. Interestingly, in the observational period reported by the authors, the histological evidence of glomerular injury started as early as 3 weeks, with podocyte effacement and progressive glomerulosclerosis. Because podocin, the podocyte-specific Cre driver used in this study to knockout Pfn1, is expressed during podocyte development, future studies using inducible knockout of Pfn1 in adult mice are warranted to confirm the requisite role of podocyte Pfn1 after kidney development.

Second, the loss of podocyte Pfn1 induced double-stranded DNA (dsDNA) damage, triggering chromatin instability and subsequent cell cycle reentry. The use of R26Fucci2aR mice, which enabled visualization of the cell phases, confirmed that podocytes in Pfn1^–/–^ mice reentered the cell cycle and committed to cellular mitosis. While the knockout of Pfn1 in podocytes altered the expression of cell cycle regulatory proteins, such as cyclins D1 and B1 as well as p21 and p53, which triggered cell cycle reentry, these podocytes underwent abortive cytokinesis and detached from the glomerular basement membrane, resulting in mitotic catastrophe (25).

Third, the authors sequenced RNA from ribosomes that were in the process of translation, via affinity purification RNA-Seq, to identify mRNAs that were actively being translated in Pfn1^–/–^ podocytes as compared with WT podocytes. Differentially expressed genes were enriched for pathways involved in RNA processing, ribonucleoprotein complex biogenesis, chromatin organization, and the cell cycle. Interestingly, among these differentially expressed genes, ribosomal RNA processing 8 (Rrp8) expression was reduced, which was conserved across all gene ontology and pathway analysis in Pfn1^–/–^ podocytes as compared with WT podocytes. Restoring Rrp8 expression in Pfn1^–/–^ podocytes attenuated the number of podocytes with dsDNA damage and mitotic catastrophe. In addition, Rrp8^–/–^ alone increased dsDNA damage and mitotic catastrophe in podocytes, demonstrating the requisite role of Pfn1/Rrp8 signaling in podocytes under basal conditions. These findings are compelling and require further investigation, as ribosomal RNA processing might provide a crucial secondary mechanism for mediating the effects of Pfn1 and eventual abortive cytokinesis in mitotic catastrophe (25).

Fourth, Tian, Pedigo, and colleagues (25) observed that glomerular Pfn1 expression was reduced in proteinuric diseases and was associated with an increase in podocytes undergoing mitotic catastrophe. However, the authors observed an increase in podocytes in the urine undergoing mitotic catastrophe primarily in patients with lupus nephritis as compared with other podocytopathies. Whether this finding is largely a result of small sample size or indicative of pathogenesis in lupus nephritis remains unclear. In addition, whether podocyte mitotic catastrophe can distinguish between primary and secondary focal segmental glomerulosclerosis remains to be investigated.

While some questions remain, this study by Tian, Pedigo, and coworkers elegantly paves the way for future investigations into the essential role of actin dynamics in mediating a fundamental mode of cell death in these postmitotic cells (25).

## Version 1 12/15/2023

Electronic publication
